# Deprexis for Veteran Depression: Open-Label Pilot Trial Examining Feasibility, Acceptability, and Preliminary Efficacy

**DOI:** 10.2196/86899

**Published:** 2026-07-21

**Authors:** Rahel Pearson, Emma Harris, Allison Metts, Christopher Graham Beevers, Paul N Pfeiffer, Suzannah Creech

**Affiliations:** 1 VISN 17 Center of Excellence for Research on Returning War Veterans, Central Texas Veterans Health Care System, 1901 Veterans Memorial Drive, Temple, TX, 76504, United States, 1 (254) 778-4811; 2 Department of Psychology, Institute for Mental Health Research, The University of Texas at Austin, Austin, TX, United States; 3 Department of Psychiatry, University of Michigan, Ann Arbor, MI, United States; 4 Department of Psychiatry and Behavioral Sciences, Dell Medical School, The University of Texas at Austin, Austin, TX, United States

**Keywords:** depression, veterans, digital intervention, pilot study, feasibility

## Abstract

**Background:**

Depression carries the highest burden of mental health–related disability in the United States. Approximately 13% of military veterans report elevated rates of depression. Despite the availability of evidence-based treatments for depression, nearly 50% of veterans in need of mental health care remain untreated. Internet-based interventions show promise in reducing this gap; however, there are currently no standard self-guided internet-based interventions for depressive symptoms in veterans. Deprexis is one such intervention that leverages cognitive behavioral therapy to target depressive symptoms.

**Objective:**

This pilot study evaluated the feasibility, acceptability, and preliminary effectiveness of Deprexis, a fully self-guided internet-based intervention for depression, in US military veterans with mild to severe depressive symptoms.

**Methods:**

This open-label pilot trial recruited 19 veterans with mild to severe depression (mean age 55.5, SD 8.2 y; baseline Quick Inventory of Depressive Symptomatology—Self-Report [QIDS-SR]: mean 16.2, SD 4.1) for an 8-week course of Deprexis, with self-report assessments at baseline, posttreatment (8 wk), and follow-up (16 wk). Primary outcomes included depressive symptoms (QIDS-SR), functional disability (World Health Organization Disability Assessment Schedule 2.0), and symptom-related disability (Sheehan Disability Scale). Feasibility was assessed through recruitment and retention rates, and acceptability was measured using validated questionnaires (Credibility and Expectancy Questionnaire and Client Satisfaction Questionnaire). Multilevel models examined change over time, with effect sizes calculated using pooled SDs from unconditional models.

**Results:**

Recruitment and retention targets were met, with 15 out of 19 (79%) participants meeting the adherence criteria (ie, ≥60 min of active program use). Of these, 14 participants completed posttreatment questionnaires and were included in the completer analyses. The program received a positive acceptability rating: of the 18 participants who completed follow-up assessments, 78% (n=14) rated services as good or excellent and 72% (n=13) were satisfied with the amount of help received. No safety concerns were reported. Among completers (n=14), QIDS-SR scores decreased from baseline to posttreatment (estimate −2.22, SE 1.44; *P*=.14; *d*=−0.54, 95% CI −1.07 to 0.13) and follow-up (estimate −2.85, SE 1.19; *P*=.02; *d*=−0.70, 95% CI −1.21 to −0.08) with moderate-to-large effect sizes. Effect sizes were similar in the total sample. Functioning (World Health Organization Disability Assessment Schedule 2.0) improved among completers at follow-up (estimate −8.09, SE 3.80; *P*=.045; *d*=−0.41, 95% CI −0.96 to −0.05). Disability (Sheehan Disability Scale) did not significantly improve from baseline to posttreatment or follow-up.

**Conclusions:**

This pilot trial demonstrates that Deprexis is feasible and acceptable for veterans with mild to severe depression, with preliminary evidence of effectiveness for depressive symptoms. The delayed emergence of functional improvements and sustained gains at follow-up support the potential of this scalable intervention. The results provide a strong foundation for the ongoing randomized controlled trial.

## Introduction

Depression is the leading cause of disability worldwide [1] and a pressing concern for veterans, who experience high rates of both lifetime and current depressive symptoms [2,3]. These symptoms are linked to diminished quality of life, impaired occupational and social functioning [4], and an increased risk of suicide [5]. Importantly, even subthreshold depressive symptoms carry a heavy burden, contributing to disability and elevating the likelihood of progression to major depressive disorder (MDD) [6-8]. Once entrenched, depressive symptoms are often chronic and recurrent [9], highlighting the need for early, accessible interventions that can interrupt this trajectory [10].

Within the Veterans Health Administration (VHA), routine depression screening creates an important opportunity to identify veterans with emerging symptoms. However, a significant treatment gap remains. Many veterans prefer psychotherapy over medication [11]; yet access to timely, evidence-based psychotherapy is limited by factors such as provider availability, stigma, and logistical barriers such as travel or competing demands [12]. Although telehealth expands access, it is resource-intensive and may not always be appropriate for veterans with milder symptoms, who nonetheless need support to prevent worsening outcomes.

Computerized interventions offer a promising solution to this treatment gap. These programs are low-cost, scalable, and accessible, while also allowing veterans to engage in treatment privately and flexibly. Evidence from community samples demonstrates that computerized interventions, including those based on cognitive behavioral approaches [13,14], mindfulness [15], and acceptance-based approaches [16], can reduce depressive symptoms and improve functioning. Deprexis, an integrative cognitive behavioral therapy (CBT)–based computerized program that draws from these therapeutic modalities, has been shown in randomized controlled trials (RCTs) to reduce depressive symptoms, improve well-being, and decrease disability among community samples [17,18]. An RCT of Deprexis (n=376) in US adults with elevated depressive symptoms demonstrated that receiving Deprexis was associated with significantly greater (*d*=.80) reductions in depressive symptoms and a 12 times greater chance of at least 50% symptom reduction, compared to the control condition [17]. Another RCT with a sample of 396 German adults found similar results: compared to the control group, participants in the treatment condition experienced significantly greater reductions in depressive symptoms (Beck Depression Inventory: odds ratio 6.8, 95% CI 2.90‐18.19) and were more likely to demonstrate clinically significant improvement (25.4% treatment group compared to 1.9% control group) [18]. However, Deprexis’ effectiveness has not been tested in veterans, who may face unique barriers to care including stigma concerns, geographic distance to services, and disabilities that limit ability to engage in care. Veterans also more frequently present with complex physical and psychiatric comorbidities that shape both engagement and outcomes.

Deprexis was selected for study in veterans based on several factors: (1) a robust evidence base in civilian populations, including meta-analytic support for its effectiveness, (2) a fully self-guided format positioned to maximize scalability and potential uptake, (3) integration of various modalities with known efficacy for depression in veterans within traditional treatment contexts, (4) an adaptive treatment format that delivers personalized content while maintaining treatment fidelity, and (5) an established US implementation structure. The data presented here were collected as part of a larger registered trial (ClinicalTrials.gov NCT06217198) designed to pragmatically evaluate Deprexis in veterans. An overview of the study protocol is described by Pearson et al [4]. The CONSORT (Consolidated Standards of Reporting Trials) checklist associated with the pilot trial is provided in Checklist 1. This trial includes both an initial pilot trial to assess feasibility, acceptability, and preliminary outcomes, as well as a subsequent, ongoing RCT to more rigorously test efficacy. The current report presents findings from the pilot trial. The aims of this pilot trial were 3-fold: (1) to evaluate the feasibility of recruiting, engaging, and retaining veterans in a fully self-guided computerized intervention; (2) to assess acceptability and credibility of the program; and (3) to explore preliminary effects on primary outcomes of depressive symptoms, functioning, and disability. These pilot data provide important insights to inform the design and implementation of the larger RCT and to determine whether Deprexis holds promise as a scalable intervention within the VHA.

## Methods

### Intervention

Deprexis is a fully self-guided, internet-based intervention for depression. While second-wave CBT is the primary theoretical orientation for Deprexis, the program also incorporates elements of third-wave CBT interventions, such as acceptance and commitment therapy and mindfulness [18]. By targeting multiple change mechanisms and offering a variety of coping strategies, Deprexis can be tailored to the user’s specific needs and preferences, potentially enhancing the treatment relevance and long-term effectiveness. The program consists of 11 interactive modules, each of which includes psychoeducation, case examples, exercises, and skill-building activities tailored to user responses. To increase motivation, Deprexis features symptom-tracking, tailored feedback, printable worksheets, audio recordings, and illustrations. The content focuses on improving depressive symptoms by addressing thoughts (eg, cognitive restructuring) and improving functioning by addressing behavioral components (eg, behavioral activation, interpersonal skills, and healthy lifestyle choices). Deprexis is designed to be flexible and adaptive, presenting personalized content based on initial inputs and choices, while maintaining a structured therapeutic flow. Modules are self-paced, with typical engagement ranging from 30 to 60 minutes per module. The details on the content of each module and Deprexis presentation are presented in Multimedia Appendix 1.

### Participants and Procedural Overview

Veterans were recruited into this pilot trial to assess feasibility, acceptability, and study procedures in advance of a larger RCT. Enrollment was completed between October 2024 and December 2024, with follow-up assessments completed by April 2025. The sample size for this study was based on feasibility recommendations for pilot trials, which suggest that 12 to 20 participants are sufficient for assessing feasibility, acceptability, and preliminary effect sizes [19].

Study procedures were as follows: (1) outreach via flyers, letters, and phone calls; (2) eligibility screening completed by veterans using the Qualtrics survey platform; (3) determination of eligibility and participant notification completed by study staff; (4) eligible participants completed and signed informed consent forms; (5) participants who signed informed consent completed the baseline questionnaire using the Qualtrics survey platform; (6) following the completion of the baseline questionnaire, participants received an email with instructions for accessing Deprexis and a unique Deprexis code; (7) an 8-week period of using Deprexis; (8) immediate posttreatment follow-up assessment using the Qualtrics survey platform; and (9) 16-week follow-up assessment using the Qualtrics survey platform.

### Recruitment and Eligibility Criteria

Veterans were recruited from a Southwest Veterans Affairs (VA) medical center through flyers in primary care and mental health clinics and via letters. Flyers included a brief description of the study, a link to the confidential online eligibility screening, and the contact number for the study coordinator, principal investigator, and local institutional review board (IRB). Letters were mailed to veterans who had a positive score on the depression clinical reminder screen (ie, Patient Health Questionnaire score>2) in the VHA electronic health record. The letter sent in the mail included (1) an introduction letter with details about the study and the phone numbers of the study coordinator, principal investigator, and the local IRB; (2) a link to the confidential online eligibility screening; and (3) the informed consent packet. Veterans who received letters and did not complete the eligibility screening or indicate disinterest were contacted by study staff via phone call to assess interest. Veterans who indicated interest over the phone were sent a link to the online eligibility screening. Disinterested or ineligible veterans were offered mental health resources and referrals. Veterans who completed the confidential online eligibility screening were contacted by study staff at which point study staff explained the study procedures and answered questions regarding the study and informed consent.

Eligible participants were required to (1) have reliable internet access (computer, tablet, or smartphone), (2) be able to provide informed consent, (3) endorse mild to severe depressive symptoms (Quick Inventory of Depressive Symptomatology—Self-Report [QIDS-SR-16] scores 6‐20; [20]), and (4) be stable on psychotropic medications for at least 30 days prior to enrollment. Participants were excluded if they (1) screened positive for one or more psychotic symptoms (score ≥1) on the Psychiatric Diagnostic Screening Questionnaire—Psychosis Subscale [21], (2) endorsed 7 or more bipolar I disorder symptoms, with at least 2 symptoms co-occurring and causing moderate to severe impairment as determined by the Mood Disorder Questionnaire [22], or (3) endorsed current suicidal intent or behavior on item-9 of the Beck Depression Inventory [23]. Final eligibility was determined by study staff (not automatically determined by the online initial eligibility screening) to ensure accurate eligibility determinations.

The QIDS-SR eligibility range (6-20) was selected to capture symptomatic veterans across the depression severity spectrum as evidence suggests that early intervention for mild depression can prevent the progression to MDD [7]. Including mild depressive symptoms enhances generalizability to real-world implementation contexts, as routine VA depression screenings identify veterans with mild depressive symptoms, who may be especially likely to benefit from Deprexis [24]. Veterans could continue to engage with all previous mental health resources for the duration of the study, as recommended for pragmatic trials.

### Feasibility and Acceptability Criteria

Feasibility and acceptability were established based on a priori benchmarks reported in digital mental health trials, including previous trials of Deprexis. Feasibility targets included: (1) enrollment of the target sample (n=16‐20) within 6 months after initiation of recruitment efforts, (2) >70% completion of follow-up assessments, (3) >70% adherence to Deprexis (ie, ≥60 min of engagement) [17], and (4) no study-related serious adverse events. In the absence of established acceptability norms for digital mental health interventions [25], acceptability was evaluated descriptively using Credibility and Expectancy Questionnaire (CEQ) and Client Satisfaction Questionnaire (CSQ) ratings.

### Procedures

Participants who met the eligibility criteria had the opportunity to ask questions and then signed the informed consent forms. The online baseline assessment was sent to participants immediately after the informed consent forms were signed. Participants completed the baseline survey an average of 4.9 (SD 4.6) days after enrolling in the study. After the baseline assessment was completed, participants received an email with instructions on how to access Deprexis and an individual Deprexis access code. Every 2 weeks, study staff received a spreadsheet from GAIA, which included participants’ total number of minutes spent on Deprexis, the date of account activation, and the most recent log-in date. These data were deidentified and linked to participants via their individual Deprexis access code. Participants who either failed to activate their Deprexis account, who demonstrated limited use (<60 min total usage), or who did not access Deprexis during the prior 2 weeks were contacted by research staff to provide reminders, answer questions, and troubleshoot access. Following the 8-week course of Deprexis, participants completed an immediate posttreatment follow-up assessment and a 16-week posttreatment follow-up assessment.

### Safety Procedures

Suicidal ideation, intent, and behavior were carefully monitored at all stages of the study. Endorsement of any level of suicidal ideation, intent, or behavior on an eligibility screening, baseline assessment, or follow-up assessment prompted an automatic pop-up with the VA suicide hotline contact information, an automatic email to the study coordinator and principal investigator, and a phone call from study staff within 1 business day of the reported suicide risk. Elevated suicide risk initiated a phone call from trained study staff who completed structured suicide risk assessments using the Columbia Suicide Severity Ratings Scale per VA suicide risk management guidelines. In the event of a positive Columbia Suicide Severity Rating Scale (ie, suicidal ideation with plan or intent or suicidal behavior in the past 3 months), a licensed clinical psychologist completed a structured Comprehensive Suicide Risk Evaluation, including a safety plan in the event of intermediate or high chronic or current risk. Participants at intermediate or high chronic or current suicide risk on the Comprehensive Suicide Risk Evaluation were referred to their local VA’s suicide prevention coordinator or nonstudy treatment as clinically indicated. No veterans endorsed intermediate or high chronic or current suicide risk at any point during study procedures. If study staff were unable to contact a participant who endorsed suicide intent or behavior, the local VA suicide prevention coordinator was contacted to initiate a wellness check. Protocol procedures adhered to the VA clinical guidelines for suicide risk assessment.

### Ethical Considerations

This study was approved by the Central Texas Veterans Health Care System (CTVHCS) IRB (2024‐001). All participants provided informed consent, with opportunities to ask questions, prior to beginning study procedures. Mental health resources and referrals were available upon request or indicated need. Participants who were excluded from the study due to psychosis symptoms, bipolar I symptoms, or very severe depressive symptoms (QIDS>20) were provided with additional mental health resources and/or a mental health referral. Additionally, data collection was HIPAA (Health Insurance Portability and Accountability Act) compliant: no personally identifiable information was collected through the online survey platform. Data were anonymized and deidentified, linked to the participant via an individual identification code. Data were collected and stored behind the VA firewall and stored on encrypted and password-protected software, as indicated on the informed consent documents. Participants were compensated US $30 for each assessment completed (ie, baseline, posttreatment, and 16-wk follow-up). Email communication with participants that included patient health or other sensitive information (eg, Deprexis access codes) was encrypted. Data on participants’ engagement with the Deprexis intervention were anonymized and deidentified and linked to the participants’ individual Deprexis access code. The protocol was amended once to allow for veterans’ names and contact details to be retained, upon participant request, in the local VA database of veterans who wish to be contacted for future participation in research studies. This amendment was made based on veterans’ requests to be considered for other research studies and was approved by the CTVHCS IRB. There were no protocol deviations. One adverse event occurred during the course of the research study (participant death due to cardiac arrest). The adverse event was reported to the CTVHCS IRB and was determined to be unrelated to the research study.

### Measures

Self-report measures of depressive symptoms, functioning, symptom-related disability, and acceptability were collected at baseline, posttreatment, and at the 16-week follow-up. Demographic information was collected at baseline only.

Depressive symptoms were assessed using the QIDS-SR-16 [20]. The QIDS-SR is a 16-item measure that captures the core symptoms of MDD. Items are rated on a 4-point scale, yielding total scores from 0 to 27, with higher scores indicating greater severity. The QIDS-SR has demonstrated strong reliability, validity, and sensitivity to change across clinical and community populations.

Functioning was measured using the World Health Organization Disability Assessment Schedule 2.0 (WHODAS 2.0) [26], a 36-item instrument evaluating functioning across 6 domains: cognition, mobility, self-care, getting along, life activities, and participation. Items are rated on a 5-point scale reflecting difficulty experienced in the past 30 days, with higher scores indicating greater impairment. The WHODAS 2.0 is widely used across health and mental health conditions and provides a comprehensive assessment of functioning.

Symptom-related disability was assessed using the Sheehan Disability Scale (SDS) [27], a 3-item measure of impairment in work/school, social life, and family/home responsibilities. Each item is rated on a 0 to 10 scale, with higher scores reflecting greater impairment. SDS total scores were prorated for participants missing 1 item at a given timepoint and total scores for participants missing 2 or more items were coded as missing. Total scores range from 0 to 30. The SDS is brief, easy to administer, and has strong psychometric support as a measure of disability [28,29].

Acceptability and intervention credibility were assessed using the CEQ [30,31] and the CSQ [32]. The CEQ is a 6-item measure that assesses participants’ perceptions of the credibility of the intervention and their expectations of benefit, using both Likert-type ratings and percentage estimates. The CSQ is an 8-item scale that evaluates satisfaction with services received, including perceived quality, helpfulness, and likelihood of recommending the program to others. Both measures are well-validated, widely used in psychotherapy and digital mental health trials, and provide complementary information about participants’ experiences with the intervention.

### Analyses

Multilevel models (MLMs) were used to examine change in outcomes (QIDS, WHODAS, and SDS) from baseline to posttreatment and follow-up. MLM was selected for 2 primary reasons. First, MLM uses all the available data under the missing-at-random assumptions, which is important given the modest attrition in this pilot trial. Second, MLM accounts for the hierarchical nature of the data, with repeated observations nested within participants. Prior to hypothesis testing, we examined variable distributions, outliers (>3 SD from mean), and missing data patterns. Baseline differences in outcomes between completers and noncompleters were examined using independent one-tailed *t* tests and chi-square analyses.

We first fit unconditional means models to establish baseline variance partitioning and calculate intraclass correlation coefficients, which quantify the proportion of total variance attributable to between-person differences compared to within-person differences. Time was then added as a categorical predictor with baseline as the reference category, allowing for the examination of specific contrasts between timepoints.

Random slope models were tested but showed poorer model fit (higher Akaike Information Criterion/Bayesian Information Criterion) compared to random intercept models. Final models, therefore, used random intercepts only to account for individual differences in baseline symptom and functional outcome levels. Likelihood ratio tests compared models with and without time effects to assess overall significance. Model assumptions were examined through residual plots, and no meaningful deviations from normality or homoscedasticity were observed. Models were fitted using restricted maximum likelihood for parameter estimation, with maximum likelihood used for model comparisons.

Cohen *d* was calculated as the model-estimated mean difference divided by the pooled SD from the unconditional models, providing standardized effect sizes that account for the longitudinal structure of the data. Effect sizes of 0.2, 0.5, and 0.8 were interpreted as small, medium, and large, respectively, following conventional benchmarks.

Two sensitivity analyses were conducted to assess the robustness of primary MLM findings. First, repeated measures ANOVA was conducted for completers for each outcome (QIDS-SR, WHODAS 2.0, and SDS), with pairwise contrasts to examine changes between specific timepoints. Second, to examine whether the quantity of program engagement predicted outcomes, Spearman correlations were computed between the total minutes of Deprexis use and change scores for each outcome at posttreatment and follow-up.

## Results

### Participants

A total of 193 veterans received a recruitment letter and at least 1 phone call, and 4 veterans indicated interest after viewing a flyer. The final sample consisted of 19 veterans with a mean age of 55.5 (SD 4.5) years and a relatively balanced sex distribution (n=10, 53% male). The majority were army veterans (n=13, 68%) with at least a bachelor’s degree (n=9, 63%). Baseline QIDS-SR scores ranged from 11 to 23; no participants scored in the mild range (6-10); 9 (47.4%) participants met the criteria for moderate depressive symptoms (11-15), and 10 (52.6%) participants met the criteria for severe depression (16-20). Participant characteristics are described in Table 1. In addition to depression, participants endorsed elevated posttraumatic stress disorder (PTSD) symptoms (PTSD checklist for DSM-5 [*Diagnostic and Statistical Manual of Mental Disorders, Fifth Edition*]: mean 40.7, SD 16.1). Three (15.8%) participants endorsed suicidal ideation at baseline, and these participants were assessed using established VA protocols (described above). No participants were excluded for active suicidal intent.

**Table 1.** Demographic characteristics of the sample (N=19).

Characteristics	Values
Age (y), mean (SD)	55.5 (4.5)
Sex: female, n (%)	9 (47.4)
Race/ethnicity, n (%)	
White, non-Hispanic	9 (47.4)
Black/African American	4 (21.1)
Asian	3 (15.8)
Multiracial	3 (15.8)
Hispanic/Latinx	1 (5.3)
Education, n (%)	
High school	1 (5.3)
Some college	6 (31.6)
Bachelor’s degree	9 (47.4)
Postgraduate	3 (15.8)
Branch of service^a^, n (%)	
Army	13 (68.4)
Navy	5 (26.3)
National Guard	2 (10.5)
Air Force/Marines	2 (10.5)
Deployment history, n (%)	
OIF/OEF/OND^b^	7 (36.8)
Other conflicts	8 (42.1)

a Branch frequency exceeds 19 participants because some participants served in multiple branches.

b OIF/OEF/OND: Operation Iraqi Freedom/Operation Enduring Freedom/Operation New Dawn.

### Feasibility

**Figure 1.**
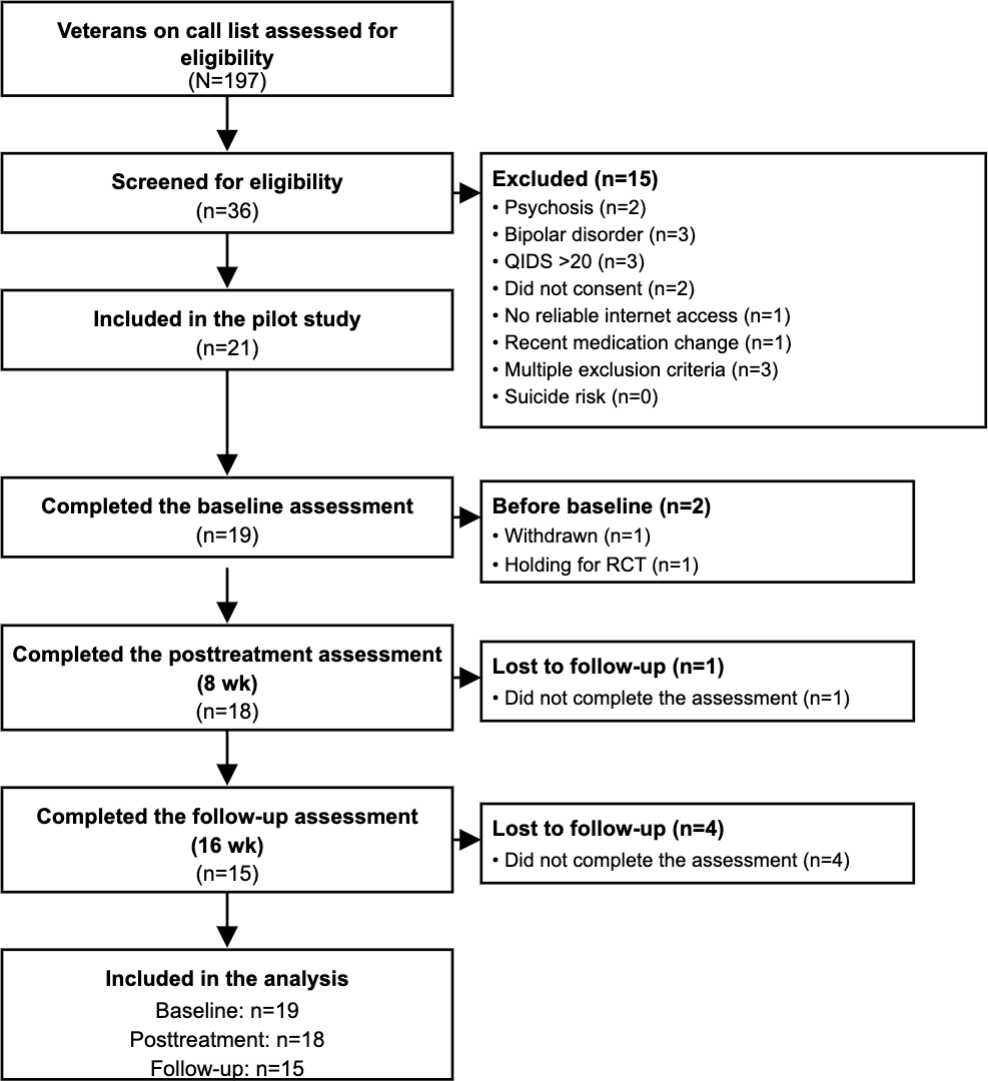
CONSORT (Consolidated Standards of Reporting Trials) diagram. QIDS: Quick Inventory of Depressive Symptomatology; RCT: randomized controlled trial.

Feasibility of trial procedures was supported by the ability to recruit the planned sample within the target time frame. Of the 197 potential participants approached through letters and phone calls, 36 were screened, and 19 consented and enrolled. Attrition was 21% (4/19); 1 participant dropped out between their baseline and posttreatment assessments and 3 additional participants dropped out between their posttreatment and follow-up assessments (Figure 1). Retention rates were therefore consistent with expectations for open trials of digital interventions [33]. There were no participants who discontinued the program due to concerns about safety or tolerability. Baseline differences between completers and noncompleters were nonsignificant across all primary outcomes (QIDS: *P*=.96; WHODAS: *P*=.43; SDS: *P*=.65), suggesting that attrition was not systematically related to initial symptom severity.

### Adherence

Adherence was defined as ≥60 minutes of total time spent on the Deprexis website over the 8-week access period (ie, 8-wk after the access code was sent via email). This is consistent with adherence cutoffs for prior Deprexis trials [17,18]. On average, participants engaged with the program for 238 (SD 216; range 20‐795) minutes, though there was substantial variability. Out of 19 participants, 15 (79%) met the ≥60 of the total program engagement adherence criteria, engaging for an average of 293 (SD 213; range 85‐795) minutes and completing a mean of 7 (SD 5.6) modules (7/11, 64%). Of these 15 participants, 14 completed posttreatment questionnaires (henceforth referred to as “completers”).

### Credibility and Acceptability

The program was rated as credible (mean 5.51, SD 1.56), and participants expected moderate benefit (mean 4.74, SD 1.55). On the CSQ (mean 23.2, SD 5), the majority endorsed positive views of the program. Out of the 18 participants who completed the posttreatment assessment, 14 (77.8%) rated the quality of services as either excellent or good, 15 (83.3%) reported that they “definitely” or “generally” received the kind of services they wanted, 17 (94.4%) indicated they would “definitely” or “generally” recommend the program to a friend in need of help, 13 (72.2%) were satisfied with the amount of help they received, 13 (72.2%) reported that the program helped them deal more effectively with their problems, 9 (50%) reported being very satisfied overall, and 14 (77.8%) stated that they would return to the program if in need of help again. Regarding needs met, 7 of 18 (38.9%) reported that the program met all of their needs, with the remainder indicating their needs were at least partially met.

### Primary Outcomes

**Figure 2.**
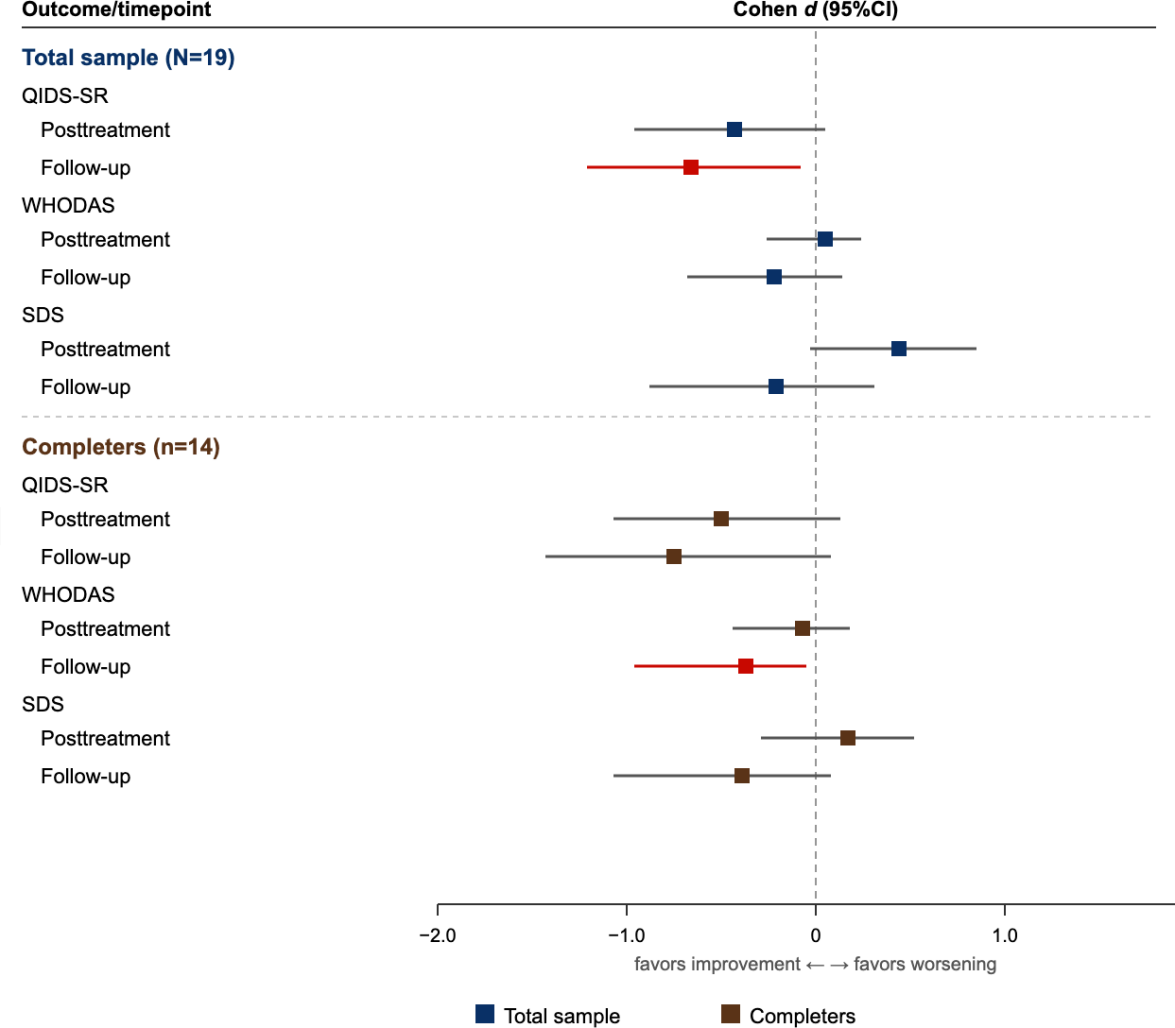
Effect sizes by outcomes and timepoints for all participants and completers. Squares represent point estimates; horizontal lines represent 95% bootstrapped CIs; QIDS-SR: Quick Inventory of Depressive Symptomatology—Self-Report; SDS: Sheehan Disability Scale; WHODAS: World Health Organization Disability Assessment Schedule 2.0.

MLMs examined change from baseline to posttreatment (8 wk) and follow-up (16 wk). Intraclass correlation coefficients ranged from 0.23 (QIDS) to 0.76 (WHODAS 2.0), indicating substantial between-person variability in outcomes. Effect sizes are presented in Figure 2. Analyses were conducted in both the full sample (N=19) and the completer sample (participants with ≥60 min of Deprexis usage who provided posttreatment data, n=14). We provide both descriptive statistics (Table 2) and standardized effect sizes, consistent with CONSORT recommendations for pilot trials.

**Table 2.** Descriptive statistics and multilevel model results by outcomes and timepoints.^a^

Measure	Baseline, mean (SD)	Posttreatment, mean (SD)	Follow-up, mean (SD)	Posttreatment vs baseline			Follow-up vs baseline		
				*Β*^b^ (SE)	*P* value	*d*^c^ (95% CI)	*Β* (SE)	*P* value	*d* (95% CI)
Total sample (N=19)									
QIDS-SR^d^	16.2 (2.5)	14.2 (4.3)	13.1 (4.9)	−1.92 (1.15)	.11	−0.47 (−0.96 to 0.05)	−2.85 (1.19)	.02^e^	−0.70 (−1.21 to −0.08)
WHODAS^f^ 2.0	83.2 (18.6)	83.2 (17.5)	74.3 (22.8)	0.25 (3.22)	.94	0.01 (−0.26 to 0.24)	−5.14 (3.45)	.15	0.26 (−0.68 to 0.14)
SDS^g^	17.3 (8.4)	21 (5.2)	15.1 (4.9)	2.80 (1.71)	.11	0.40 (−0.03 to 0.85)	−1.75 (1.75)	.33	−0.25 (−0.88 to 0.31)
Completers (n=14)									
QIDS-SR	16.7 (2.6)	14.5 (4.3)	13.3 (15.2)	−2.22 (1.44)	.14	−0.54 (−1.07 to 0.13)	−3.23 (1.56)	.05	−0.79 (−1.43 to 0.08)
WHODAS 2.0	87.9 (17.9)	85.7 (18.6)	75.4 (22.0)	−2.25 (3.35)	.51	−0.11 (−0.44 to 0.18)	−8.09 (3.80)	.045	−0.41 (−0.96 to −0.05)
SDS	18.6 (8.9)	20.8 (5.5)	14.4 (4.4)	0.90 (1.63)	.59	−0.13 (−0.29 to 0.52)	−3.00 (1.75)	.10	−0.43 (−1.07 to 0.08)

a 95% CI values are bootstrapped (1000 iterations). Completers defined as participants with ≥60 minutes of Deprexis use who provided posttreatment data.

b *Β*: unstandardized regression coefficient.

c *d*: Cohen *d* calculated from the pooled SD of unconditional model.

d QIDS-SR: Quick Inventory of Depressive Symptomatology—Self-Report.

e *P*<.05.

f WHODAS 2.0: World Health Organization Disability Assessment Schedule 2.0.

g SDS: Sheehan Disability Scale.

#### Depression (QIDS*)*

Likelihood ratio tests revealed a significant overall time effect (*χ*²_2_=13.84; *P*=.02). In the total sample, QIDS scores showed a nonsignificant reduction from baseline to posttreatment (estimate −1.92, SE 1.15; *P*=.11; Cohen *d*=−0.47, 95% CI −0.96 to 0.05). From baseline to follow-up, improvements were statistically significant with moderate effect sizes (estimate −2.85, SE 1.19; *P*=.02; *d*=−0.70, 95% CI −1.21 to −0.08). Among completers (n=14), baseline-to-post reductions were similar (estimate −2.22, SE 1.44; *P*=.14; *d*=−0.54, 95% CI −1.07, 0.13), while baseline-to-follow-up reductions approached significance with large effect sizes (estimate −3.23, SE 1.56; *P*=.05; *d*=−0.79, 95%CI −1.43 to 0.08).

#### Functioning (WHODAS 2.0)

The overall time effect was nonsignificant (*χ*²_2_=2.99; *P*=.22). For the total sample, WHODAS 2.0 scores showed minimal change from baseline to posttreatment (estimate 0.25, SE 3.22; *P*=.94; *d*=0.01, 95% CI −0.26 to 0.24) and nonsignificant trends toward improvement at follow-up (estimate −5.14, SE 3.45; *P*=.15; *d*=−0.26, 95% CI −0.68 to 0.14). However, completers demonstrated significant moderate improvement from baseline to follow-up (estimate −8.09, SE 3.80; *P*=.045; *d*=−0.41, 95% CI −0.96 to −0.05).

#### Disability (SDS)

Likelihood ratio tests revealed a significant overall time effect (*χ*²_2_=7.33; *P*=.03). In the total sample, SDS scores showed a nonsignificant worsening from baseline to posttreatment (estimate 2.80, SE 1.71; *P*=.11; *d*=0.40, 95% CI −0.03 to 0.85), followed by nonsignificant improvement from baseline to follow-up (estimate −1.75, SE 1.75; *P*=.33; *d*=−0.25, 95% CI −0.88 to 0.31). Among completers, patterns were similar to temporary worsening at posttreatment, followed by a nonsignificant trend toward improvement at follow-up (estimate −3.00, SE 1.75; *P*=.10; *d*=−0.43, 95% CI −1.07 to 0.08).

#### Sensitivity Analyses

To assess the robustness of MLM findings, complete cases of repeated measures ANOVA were conducted for the 3 outcomes. For QIDS-SR, there was a significant effect of time (*F*_2,24_=3.79; *P*=.04), with contrasts indicating a significant reduction from baseline to posttreatment (*t*_12_=2.26; *P*=.05) and a trending reduction from baseline to follow-up (*t*_12_=2.10; *P*=.06). There was no significant effect of time on WHODAS 2.0 scores (*F*_2,26_ = 1.12; *P*=.34). For SDS, there was a trending effect of time (*F*_2,18_ = 3.18; *P*=.07), with a significant improvement from posttreatment to follow-up (*t*_9_=3.17; *P*=.01). Overall, the results of the ANOVA analyses were consistent with the MLM analyses across the 3 outcomes. We examined engagement as a continuous variable using Spearman correlations between total minutes of program use with change across QIDS-SR, WHODAS 2.0, and SDS scores at posttreatment and follow-up. There was no significant dose-response relationship for any of the examined outcomes, suggesting that engagement threshold may be more clinically meaningful than quantity of use. This is broadly consistent with the broader literature, which suggests that continuous engagement metrics do not consistently predict outcomes in digital intervention trials [34].

## Discussion

### Principal Results

This pilot trial provides preliminary evidence to support the feasibility, acceptability, and potential effectiveness of Deprexis for veterans presenting with mild to severe depressive symptoms. The successful recruitment and retention of participants within the target time frame demonstrate that veterans are willing to engage with fully self-guided digital interventions for depression. Adherence rates in this study (15/19, 79%, meeting the 60-min threshold) compare favorably to adherence rates reported in previous trials of digital CBT interventions [33,35]. This finding is particularly encouraging, given that our intervention required no therapist contact or peer support, unlike other successful programs that have incorporated such elements for veteran populations [36].

The high credibility ratings and positive acceptability scores suggest that veterans view the intervention as a legitimate treatment option. The absence of safety concerns is particularly important given that veterans are considered a higher-risk population for adverse outcomes, including suicidal ideation, especially in the context of depression [37]. The pattern of outcomes observed in this pilot trial suggests some potential benefits, though interpretation must be cautious given the small sample size and mixed findings. While depressive symptoms showed improvement at posttreatment and follow-up with moderate-to-large effect sizes (*d*=−0.47 to *d*=−0.79), the temporary worsening of disability scores at posttreatment followed by recovery at follow-up indicates a complex pattern that requires replication in a larger, controlled study. The maintenance of depressive symptom improvements at 16-week follow-up is particularly noteworthy, as many digital interventions show attenuation of effects over time [38]. The pattern observed in the total sample, where improvement in depressive symptoms was more pronounced at follow-up than at posttreatment, may be attributed to incremental gains due to additional skills practice, natural recovery, or a combination thereof. The negligible change from posttreatment to follow-up suggests that gains achieved during the 8-week intervention period were sustained, which is consistent with findings from longer-term follow-up studies of Deprexis in civilian populations [18].

This sample had a high prevalence of significant PTSD symptomatology, which reflects a well-documented comorbidity between PTSD and depression in veterans [39]. Depression is this study’s primary outcome, given the scope of our intervention, as well as the functional burden and suicide risk associated with depressive symptoms in veterans. However, depressive symptoms and PTSD share treatment mechanisms included in Deprexis (eg, behavioral activation and cognitive restructuring). The full RCT of Deprexis for veterans will examine the moderating influence of PTSD on depressive symptoms and the effects of Deprexis on PTSD symptoms. The delayed emergence of functional improvements aligns with findings that functional recovery often lags behind symptomatic improvement in depression treatment [40]. The moderate effect size for functional improvement among treatment completers (*d*=−0.41) suggests that veterans who engage meaningfully with the intervention may experience clinically meaningful improvements in disability and daily functioning. This finding is particularly relevant given that functional impairment is often a primary concern for veterans seeking mental health treatment and is strongly associated with quality-of-life outcomes [41].

### Comparison With Prior Work

These pilot trial findings contribute to a growing evidence base supporting the effectiveness of digital mental health interventions for veteran populations. Our recent systematic review of computerized psychological interventions in veterans and service members identified significant benefits for various mental health conditions, though studies specifically targeting depression have been limited [42]. The effect sizes for depressive symptoms observed in this pilot study are comparable to effect sizes reported in meta-analyses of Deprexis trials [43], though such comparisons should be interpreted with considerable caution given the large SEs, lack of control group, and small sample size in the current pilot trial.

The high credibility ratings and positive acceptability scores align with previous evaluations of Deprexis in civilian populations [17,18] and suggest that veterans view the intervention similarly to civilian samples. These findings also align with results from peer-supported computerized CBT studies that have shown modest but clinically meaningful improvements in depression symptoms among veterans in primary care settings [36]. The positive feasibility and acceptability findings support the potential for scalable implementation of Deprexis within VHA settings. The minimal resource requirements of a fully self-guided intervention make it particularly attractive for addressing the treatment gap between the demand for mental health services and provider availability within the VHA system [12]. The ability to engage veterans privately and flexibly may be particularly valuable for those who face barriers to traditional face-to-face therapy, including stigma concerns and logistical challenges [12].

Treatment cost is an important consideration for implementation planning. Study participants received access to Deprexis at no charge; however, cost may be a barrier for veterans who pay out-of-pocket to access the intervention. For VHA implementation, agency-level licensing agreements may be negotiated to significantly reduce the cost of Deprexis, and cost-effectiveness analyses will be an essential component of future implementation decisions.

### Limitations

Several important limitations must be acknowledged that temper the interpretation of these preliminary findings. The open-trial design without a control group limits our ability to attribute observed changes definitively to the intervention, and the small sample size means that effect size estimates are unstable and should be interpreted with caution. The reliance on self-report measures introduces potential bias, and the absence of clinician-rated outcomes limits our ability to assess the clinical significance of observed changes. This concern is attenuated by results from a recent meta-analysis of 91 RCTs of psychotherapy for depression, which found that treatment effects from self-report and clinician-rated instruments are highly comparable [44]. Attrition, while modest, may have introduced selection bias favoring participants who were more engaged or experiencing greater benefit from the intervention. Recruitment via outreach letters targeting individuals with a recent positive depressive symptom screen is a pragmatic approach that aligns with real-world VA implementation. The overall recruitment yield of 9.6% (19/197) is consistent with outreach-based recruitment approaches, where contact with individuals not explicitly help-seeking is expected to yield lower response rates than self-referred samples [42]. However, the selection bias inherent in this recruitment method and the sample’s demographic characteristics, including high educational attainment, middle age, and mild to severe depression, may limit generalizability to the broader veteran population. Future research should examine Deprexis’ effectiveness across diverse veteran subgroups and explore factors that predict engagement and outcomes in this population.

The use of fully self-guided digital interventions raises important ethical considerations, especially regarding suicide risk triaging. This trial employed a multilayered safety strategy, which included automated risk monitoring during assessments, structured suicide assessment protocols, appropriate clinical triaging, and continued access to standard VA care. The absence of any study-related adverse events and the successful management of veterans with suicidal ideation suggest that our risk mitigation approach was sufficient for veterans with mild to severe depression. However, self-guided digital interventions may not be appropriate for all veterans. Future trials and implementation efforts should prioritize evidence-informed screening approaches and clear escalation pathways to higher levels of care.

As a pilot trial, our primary objectives were the assessment of feasibility, acceptability, and preliminary effect estimation, not definitive hypothesis testing. These pilot findings support proceeding to a randomized evaluation of Deprexis in veterans while maintaining appropriate scientific caution about claims of clinical effectiveness.

### Conclusions

The findings of this pilot trial provide a strong foundation for the larger RCT that is currently underway. These findings informed several procedural modifications, including enhanced outreach strategies for participants and increased monitoring of engagement patterns. These adaptations reflect the importance of optimizing implementation procedures based on real-world feasibility data, particularly for digital interventions where engagement patterns can vary substantially across different populations [13,14]. Feasibility and acceptability findings reported in this pilot study are encouraging and support moving forward with an RCT. Claims about clinical effectiveness appear to be favorable but must await replication with adequate statistical power and appropriate control conditions. Innovative, evidence-based digital interventions represent an important component of a comprehensive care continuum that can adapt to the complex clinical needs of veterans. The promising preliminary results reported here suggest that Deprexis may help bridge the treatment gap for veterans who might otherwise go without adequate mental health care.

## Supplementary material

10.2196/86899 Multimedia Appendix 1 Deprexis module content and presentation.

10.2196/86899 Checklist 1 CONSORT checklist.

## Abbreviations

CBT: cognitive behavioral therapy
CEQ: Credibility and Expectancy Questionnaire
CONSORT: Consolidated Standards of Reporting Trials
CSQ: Client Satisfaction Questionnaire
CTVHCS: Central Texas Veterans Health Care System
HIPAA: Health Insurance Portability and Accountability Act
IRB: Institutional Review Board
MDD: major depressive disorder
MLM: multilevel model
PHQ-2: Patient Health Questionnaire-2
PTSD: posttraumatic stress disorder
QIDS-SR-16: Quick Inventory of Depressive Symptomatology—Self-Report (16-item)
RCT: randomized controlled trial
SDS: Sheehan Disability Scale
VA: Veterans Affairs
VHA: Veterans Health Administration
WHODAS 2.0: World Health Organization Disability Assessment Schedule 2.0
