# Supplementary material for: Deprexis for Veteran Depression: Open-Label Pilot Trial Examining Feasibility, Acceptability, and Preliminary Efficacy
Source: JMIR Form Res. 2026 Jul 21;10:e86899. doi: 10.2196/86899 (PMC13387593; doi:10.2196/86899)
Supplement: Multimedia Appendix 1 [file formative-v10-e86899-s001.docx]

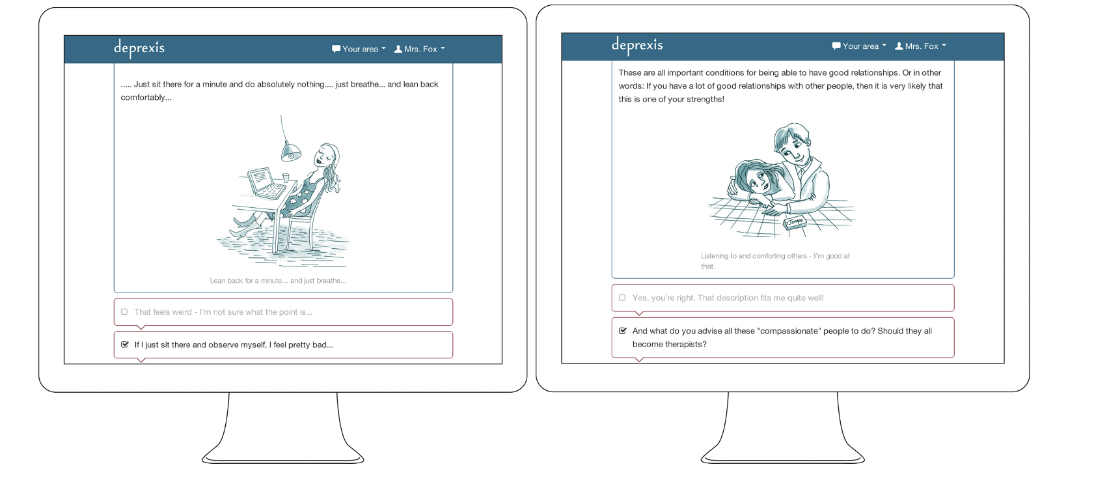


| **Module** | **Content** |
| --- | --- |
| Introduction | Overview of Deprexis program; psychoeducation about the relationships between thoughts, feelings, and behaviors; mindfulness exercise; worksheet to encourage regular program use |
| Behavioral activation | Activity-mood relationship; psychoeducation on basic psychological needs; selecting and scheduling activities using a checklist and log; problem-solving barriers to activity completion |
| Cognitive modification | Psychoeducation on automatic thoughts; cognitive distortions; strategies for cognitive restructuring (e.g., birds-eye view, scientist perspective) |
| Relaxation, physical exercise, and lifestyle modification | Relationship between lifestyle habits (e.g., sleep, exercise, diet) and depression; instruction and practice of relaxation techniques (e.g., diaphragmatic breathing, visual imagery) |
| Acceptance and mindfulness | Accepting unwanted thoughts and feelings using metaphors and exercises; mindfulness exercises (e.g., leaves on a stream); values clarification and committed action |
| Problem solving | Defining problems in concrete terms; setting achievable goals; generating and evaluating potential solutions; implementing and reviewing outcomes |
| Childhood experiences | Processing difficult childhood memories; expressive writing; forming new positive memories; forgiveness |
| Interpersonal skills | Interpersonal functioning and depression; communication styles (e.g., assertive, passive-aggressive, non-verbal); practicing non-blaming communication techniques |
| Positive psychology | Strengths-based approach to well-being; savoring positive experiences; identifying and fostering personal talents |
| Dreamwork and emotion-focused intervention | Coping with distressing dreams (e.g., dream journaling, rewriting dream endings); reconceptualizing dreams as a problem-solving mechanism |
