# Supplementary material for: Deprexis for Veteran Depression: Open-Label Pilot Trial Examining Feasibility, Acceptability, and Preliminary Efficacy
Source: JMIR Form Res. 2026 Jul 21;10:e86899. doi: 10.2196/86899 (PMC13387593; doi:10.2196/86899)
Supplement: Checklist 1 [file formative-v10-e86899-s002.pdf]

CONSORT 2010 Checklist Extension for Pilot and Feasibility Trials

Manuscript Title: A Pilot Evaluation of Deprexis: Feasibility, Acceptability, and Preliminary Efficacy in a Veteran Sample

Authors: Rahel Pearson, Emma Harris, Allison Metts, Christopher G. Beevers, Paul N. Pfeiffer, Suzannah K. Creech

Note: This is an open-label (non-randomized) pilot trial; therefore, items 8a-11b regarding randomization and blinding are marked as Not Applicable (N/A).

TITLE AND ABSTRACT

Item	Checklist Item	Reported on Page
**1a**	Identification as a pilot or feasibility trial in the title	Page 1
**1b**	Structured summary of pilot trial design, methods, results, and conclusions	Page 1

INTRODUCTION

Item	Checklist Item	Reported on Page
**2a**	Scientific background and explanation of rationale for future definitive trial, and reasons for pilot trial	Pages 2-3
**2b**	Specific objectives or research questions for pilot trial	Page 3

METHODS

Trial Design

Item	Checklist Item	Reported on Page
**3a**	Description of pilot trial design including allocation ratio	Pages 1, 4
**3b**	Important changes to methods after pilot trial commencement, with reasons	N/A - No changes made

Participants

Item	Checklist Item	Reported on Page
**4a**	Eligibility criteria for participants	Page 4
**4b**	Settings and locations where the data were collected	Pages 4-5
**4c**	How participants were identified and consented	Pages 4-5

Interventions

Item	Checklist Item	Reported on Page
**5**	The interventions for each group with sufficient details to allow replication, including how and when they were actually administered	Pages 5-6

Outcomes

Item	Checklist Item	Reported[illegible]
**6a**	Completely defined prespecified assessments or measurements to address each pilot trial objective, including how and when they were assessed	Pages 5-7
**6b**	Any changes to pilot trial assessments or measurements after commencement, with reasons	N/A - No ch[illegible]
**6c**	If applicable, prespecified criteria used to judge whether, or how, to proceed with future definitive trial	N/A

Sample Size

Item	Checklist Item	Reported on Page
**7a**	Rationale for numbers in the pilot trial	Page 4
**7b**	When applicable, explanation of any interim analyses and stopping guidelines	N/A - No interim analyses

Randomisation

Item	Checklist Item	Reported on Page
**8a**	Method used to generate the random allocation sequence	N/A - Open-label pilot trial
**8b**	Type of randomisation; details of restrictions	N/A - Open-label pilot trial
**9**	Mechanism used to implement the random allocation sequence	N/A - Open-label pilot trial
**10**	Who generated sequence, enrolled participants, assigned interventions	N/A - Open-label pilot trial

Blinding

Item	Checklist Item	Reported on Page
**11a**	If done, who was blinded after assignment to interventions	N/A - Open-label design
**11b**	If relevant, description of the similarity of interventions	N/A - Single intervention group

Statistical Methods

Item	Checklist Item	Reported on Page
**12**	Methods used to address each pilot trial objective whether qualitative or quantitative	Pages 7-8

RESULTS

Participant Flow

Item	Checklist Item
**13a**	For each group, the numbers of participants who were approached and/or assessed for eligibility, enrolled, received intended treatment, and were assess[illegible]
**13b**	For each group, losses and exclusions after enrollment, together with reasons

Recruitment

Item	Checklist Item	Reported on Page
**14a**	Dates defining the periods of recruitment and follow-up	Page 4
**14b**	Why the pilot trial ended or was stopped	Page 9

Baseline Data

Item	Checklist Item	Reported on Page
**15**	A table showing baseline demographic and clinical characteristics for each group	Pages 8-9; Table 1 (Page 9)

Numbers Analysed

Item	Checklist Item	Reported on Page
**16**	For each objective, number of participants (denominator) included in each analysis	Pages 12-14

Outcomes and Estimation

Item	Checklist Item	Reported on Page
**17**	For each objective, results including expressions of uncertainty (such as 95% confidence interval) for any estimates	Pages 12-14; Figure 2 (Page 13)

Ancillary Analyses

Item	Checklist Item	Reported on Page
**18**	Results of any other analyses performed that could be used to inform the future definitive trial	Pages 11-12

Harms

Item	Checklist Item	Reported on Page
**19**	All important harms or unintended effects in each group	Page 10
**19a**	If relevant, other important unintended consequences	Page 10

DISCUSSION

Item	Checklist Item	Repo[illegible]
**20**	Pilot trial limitations, addressing sources of potential bias and remaining uncertainty about feasibility	Page[illegible]
**21**	Generalisability (applicability) of pilot trial methods and findings to future definitive trial and other studies	Page[illegible]
**22**	Interpretation consistent with pilot trial objectives and findings, balancing potential benefits and harms, and considering other relevant evidence	Page[illegible]
**22a**	Implications for progression from pilot to future definitive trial, including any proposed amendments	Page[illegible]

OTHER INFORMATION

Item	Checklist Item	Reported on Page
**23**	Registration number for pilot trial and name of trial registry	Page 3 (ClinicalTrials.gov NCT06217198)
**24**	Where the pilot trial protocol can be accessed, if available	Page 3 (Pearson et al., 2024, JMIR Research Protocols)
**25**	Sources of funding and other support, role of funders	Page 18
**26**	Ethical approval or approval by research review committee, confirmed with reference number	Page 5 (IRB #2024-001)
